# Classification of Thyroid Nodules with Stacked Denoising Sparse Autoencoder

**DOI:** 10.1155/2020/9015713

**Published:** 2020-12-07

**Authors:** Zexin Li, Kaiji Yang, Lili Zhang, Chiju Wei, Peixuan Yang, Wencan Xu

**Affiliations:** ^1^Health Care Center, The First Affiliated Hospital of Shantou University Medical College, No. 57, Changping Road, Shantou 515041, China; ^2^Department of Radiology, The First Affiliated Hospital of Shantou University Medical College, No. 57, Changping Road, Shantou 515041, China; ^3^Multidisciplinary Research Center, Shantou University, No. 243, Daxue Road, Shantou 515063, China; ^4^Department of Endocrinology, The First Affiliated Hospital of Shantou University Medical College, No. 57, Changping Road, Shantou 515041, China

## Abstract

**Purpose:**

Several commercial tests have been used for the classification of indeterminate thyroid nodules in cytology. However, the geographic inconvenience and high cost confine their widespread use. This study aims to develop a classifier for conveniently clinical utility.

**Methods:**

Gene expression data of thyroid nodule tissues were collected from three public databases. Immune-related genes were used to construct the classifier with stacked denoising sparse autoencoder.

**Results:**

The classifier performed well in discriminating malignant and benign thyroid nodules, with an area under the curve of 0.785 [0.638–0.931], accuracy of 92.9% [92.7–93.0%], sensitivity of 98.6% [95.9–101.3%], specificity of 58.3% [30.4–86.2%], positive likelihood ratio of 2.367 [1.211–4.625], and negative likelihood ratio of 0.024 [0.003–0.177]. In the cancer prevalence range of 20–40% for indeterminate thyroid nodules in cytology, the range of negative predictive value of this classifier was 37–61%, and the range of positive predictive value was 98–99%.

**Conclusion:**

The classifier developed in this study has the superb discriminative ability for thyroid nodules. However, it needs validation in cytologically indeterminate thyroid nodules before clinical use.

## 1. Introduction

Thyroid nodules (TNs) are the most frequently encountered thyroid diseases and show an escalating prevalence in recent years. By neck palpation, 4% to 7% of adults are found with TNs [1]. By sensitive imaging apparatus, such as the ultrasonic diagnostic system, 13% to 68% of patients are diagnosed with TNs [2, 3]. Most of the TNs are detected incidentally in a health checkup or an examination for other head and neck diseases. Despite the high prevalence of TNs, most of them are benign. Nevertheless, it is reported that 5%–13% of TNs are with a high risk of malignancy [4].

Once the nodules are suspected as malignant by ultrasound, fine-needle aspiration (FNA) cytology is a wise choice to assess the TNs and produce a risk stratification [5]. Fortunately, approximately 55–74% of FNA samples are diagnosed as benign nodules (Bethesda II), and only 2–5% of FNA samples are diagnosed as malignant nodules (Bethesda VI). Nevertheless, the remaining samples are classified into indeterminate thyroid nodules (ITNs), including 2–18% of the nodules in Bethesda III, 2–25% in Bethesda IV, and 1–6% in Bethesda [6]. Once the TNs are diagnosed as indeterminate, most patients are referred to surgical treatment. However, 69% of the ITNs are diagnosed as benign by postsurgical pathology [7]. This indicates 69% of the patients with ITNs have unnecessary surgeries, and some of them have to take the lifetime thyroxine supplementation. As well, unnecessary surgeries will cause an overload of medical expenditure [8].

Nowadays, several commercial molecular tests are invented sequentially to make a more precise preoperative diagnosis for TNs and prevent unnecessary surgery. As we know, there are five commercially available molecular tests, Afirma, ThyroSeq v2, ThyroSeq v3, ThyGenX/ThyraMIR, and RosettaGXReveal [9–13], which have benefited patients with ITNs. These molecular tests have been demonstrated to perform outstandingly, despite the different sensitivity, specificity, positive predictive values (PPVs), and negative predictive values (NPVs). However, access to these molecular tests is geographically inconvenient and economically unaffordable for some patients [14]. In this study, based on stacked denoising sparse autoencoder (SDSAE), we developed a classifier using TNs samples tested by multiple RNA-testing platforms.

## 2. Methods

### 2.1. Study Design and Subjects

Gene expression data of TNs were collected from three major databases, The Cancer Genome Altas (TCGA), ArrayExpress, and Gene Expression Omnibus (GEO). Two RNA-sequencing and seven microarray datasets were eligible and enrolled in this study (Table 1). We only chose the interesting samples of thyroid nodular lesions, so 1013 tissue samples were included for further analysis. The pathological diagnosis of the tissue samples is presented in Table 2. There were two data types in these databases, processed and raw data. The processed data are background-subtracted and within-platform normalized. For the convenience of data analysis, we preferentially downloaded the processed data, such as Fragments per Kilobase Million (FPKM) in TCGA, series matrix files in GEO, and processed data in ArrayExpress. Some genes, detected by several probes, had several values. We chose the median value. Datasets E-MEXP-97 and GSE33630 were picked up as the independent testing set (*n* = 84), and the rest datasets were combined as the training set (*n* = 929). The study was approved by the Ethics Committee of the First Affiliated Hospital of Shantou University Medical College.

### 2.2. Construction of the Classifier Based on the Stacked Denoising Sparse Autoencoder

Carcinoma is closely related to immune cell infiltration in its microenvironment. Some immune signatures have been developed as prognostic predictors [15]. It is feasible to use the immune-related genes (IRGs) to discriminate benign and malignant TNs. One-thousand eight-hundred and eleven IRGs were downloaded from the ImmPort Shared Data (https://www.immport.org/shared/home). The biological function of IRGs is diverse, including antigen processing and presentation, BCR signaling pathway, TCR signaling pathway, natural-killer cell cytotoxicity, antimicrobials, chemokines, chemokine receptors, cytokines, and cytokine receptors. Nine-hundred and twenty-three IRGs, measured by all the platforms of the datasets, were chosen for further analysis. Since our data came from different platforms, it was hard to analyze them without normalization and removal of the batch effect. We used a pairwise scoring system, which was well performed and validated in the previous study [16] to solve this problem. We used every two different genes of the 923 IRGs to create a gene pair (genes I and II), so each sample had 425503 gene pairs as its features. Then, we scored each gene pair by comparing the expression levels of gene I and gene II. When gene I > gene II, this gene pair was scored as one; otherwise, the score was zero. This scoring approach, based on the comparison of two IRGs, has an advantage that we can gather samples from different platforms and analyze them together regardless of the normalization and batch effect, which are necessary procedures before analyzing genomic data. The shortage is that the feature number of one sample is expanded exponentially, from 923 to 425503, resulting in a hugely increased computation. To reduce the feature number of 425503 that was used to construct the classifier, we used correlation analysis to remove the features less correlated to the classification of the TNs. Nine-thousand three hundred and seventy-seven features with correlation coefficient ≥0.4 and *P* value < 0.05 were retained. These 9377 features, namely, gene pairs, consisted of 897 IRGs (supplementary information).

Autoencoder (AE) is an unsupervised artificial neural network. It consists of two processes, encoding, and decoding. The encoding process encodes the input data into efficient data codings, which can be decoded to reconstruct the input data in the decoding process. During the encoding and decoding, some datum noises can be removed from the original data. This advantage renders the autoencoder become a feature extraction tool. Besides, the autoencoder is usually used to learn a data representation, which is usually a smaller size than the original data. Thus, another advantage of the autoencoder is to reduce the data dimensionally to save calculating time.

Denoising sparse autoencoder (DSAE), which adds corruption operation and sparsity constraint into the traditional autoencoder, can extract more robust and useful features. The corruption operation sets some of the input data to zero, and the autoencoder tries to undo the effect of the corruption operation. This harsh learning process helps the autoencoder to learn more information about the input data. The sparsity constraint is imposed on the hidden layers, which will constrain the neurons to be inactive most of the time. This also helps the autoencoder to discover more interesting structures of the data. The parameters of corruption and sparsity were set as 0.5 and 0.1, respectively, in this study.

**Figure 1:**
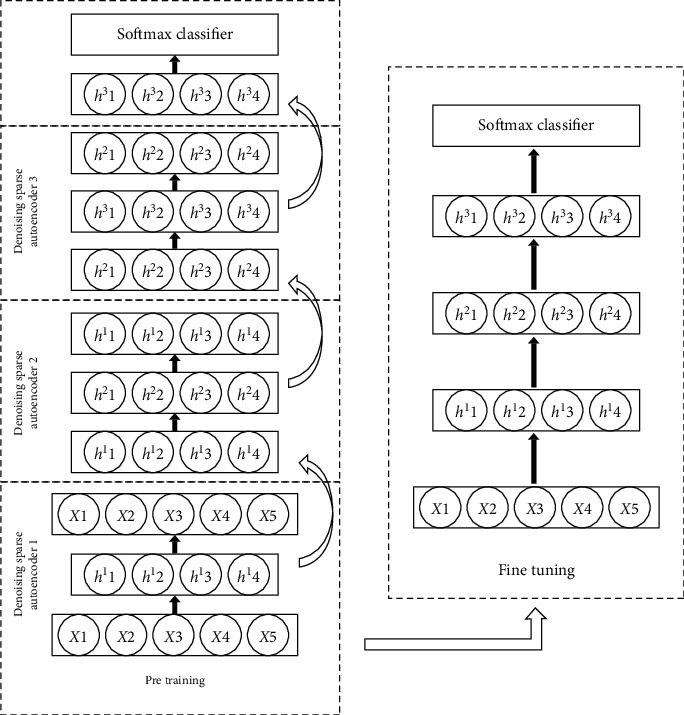
Training procedure of the immune-related classifier. The training of the immune-related classifier was based on stacked denoising sparse autoencoder, including two phrases, pretraining and fine-tuning.

A stacked denoising sparse autoencoder (SDSAE) is a deep neural network. It is stacked up with the hidden layer of several DSAE. Details about SDSAE are elaborated in the previous study [17]. Here, we briefly interpret the structure we used in this study. As Figure 1 shows, the training of an SDSAE includes two phrases, pretraining and fine-tuning. In pretraining, each DSAE is trained separately and sequentially with the backpropagation algorithm to minimize the loss. After the training of the first DSAE, the hidden layer (20 neurons) is extracted to feed into the second DSAE, and then the hidden layer (20 neurons) of the second DSAE is extracted to feed into the third DSAE. Finally, the hidden layer (20 neurons) of the third DSAE is used to connect to a softmax classifier for the supervised training. In fine-tuning, after all the hidden layers and the softmax classifier are stacked up, the whole network is trained to improve the classification ability based on the weights in the pretraining. In this study, we used an SDSAE to construct an immune-related classifier (IRC). To prevent the overfitting, we used 80% of the samples of the training set to train the model and 20% as validation to make an early stopping.

Machine learning has played an essential role in a broad range of important applications, especially in prediction and classification. It contains numerous algorithms. Each algorithm has its strengths and weaknesses in different data types. We also tried to develop classifiers with other four powerful algorithms, multilayer perception, logistic regression, random forest, and support vector machine.

### 2.3. Statistical Analysis

*Python* (version 3.7) and *R* (version 3.5.3) were used for the statistical analysis.In python, SDSAE was performed in the environment Tensorflow. The codes were referred to and modified based on the previous study (https://github.com/wblgers/tensorflow_stacked_denoising_autoencoder). MLPclassifier, logisticregression, RandomForestClassifier, and SVC were imported from sklearn for neural network, logistic regression, random forest, and support vector machine algorithm, respectively. The main parameters of these four algorithms were as follows: MLPClassifier (hidden_layer_sizes = (200, 100), solver = “sgd”, early_stopping = True, alpha = 0.1, validation_fraction = 0.20); LogisticRegression (solver = “lbfgs”); RandomForestClassifier (*n*_estimators = 200, max_depth = 7, max_features = 10); SVC (kernel = “linear”, *C* = 0.1). In *R*, cor function was used to calculate the correlation coefficient. Package reportROC was used to calculate the area under the curve (AUC), accuracy, sensitivity, specificity, positive likelihood ratio (PLR), negative likelihood ratio (NLR), positive predictive value (PPV), and negative predictive value (NPV). Microsoft Word 2010 was used for plotting.

### 2.4. Limitations of the Study

Since data from this study relied upon public databases that only reported the postoperative pathologies, the cytological classifications of the ITNs were unavailable for conducting IRC analysis. However, a previous commercial classifier, ThyroSeq v3, was developed based on 238 tissue samples, and it was demonstrated to have a high accuracy of 90.9% in 175 FNA samples of ITNs. Thus, our classifier likely performs similarly well in the FNA samples of ITNs, since it was also developed based on the tissue samples.

## 3. Results

### 3.1. Performance of the IRC Based on SDSAE

In training set, the IRC had a good performance, with an AUC of 0.887 [0.855–0.920], accuracy of 92.0% [92.0–92.0%], sensitivity of 93.4% [91.6–95.1%], specificity of 84.1% [77.9–90.3%], PLR of 5.868 [3.962–8.689], and NLR of 0.079 [0.060–0.104]. The PPV and NPV were 97.3% [96.1–98.4%] and 67.7% [60.5–74.8%], respectively, when the malignancy rate was 85.8%. In testing set, the IRC performed as well as in training set, with an AUC of 0.785 [0.638–0.931], accuracy of 92.9% [92.7–93.0%], sensitivity of 98.6% [95.9–101.3%], specificity of 58.3% [30.4–86.2%], PLR of 2.367 [1.211–4.625], and NLR of 0.024 [0.003–0.177]. The PPV and NPV were 93.4% [87.8–99%] and 87.5% [64.6–110.4%], respectively, when the malignancy rate was 85.7% (Table 3). The accuracies of IRC on different pathologies of TNs were presented in Table 4. In the independent testing set, the accuracies of IRC on different pathologies of TNs were 100% in anaplastic and papillary thyroid carcinoma, 91.7% in follicular thyroid carcinoma, and 58.3% in follicular thyroid adenoma.

We compared the IRC with the classifiers developed by four powerful algorithms, multilayer perception, logistic regression, random forest, and support vector machine. Unfortunately, multilayer perception and random forest algorithms failed in the independent testing set. They could not recognize the benign TNs and classified all the benign TNs into malignancy. Logistic regression and support vector machine as well did not perform ideally in recognizing the benign TNs with low specificity of 16.7%, although they had a high sensitivity of 98.6% (Table 5).

### 3.2. Comparisons the IRC with Five Commercial Molecular Tests

Afirma, ThyroSeq v2, ThyroSeq v3, ThyGenX/ThyraMIR, and RosettaGX Reveal are five molecular tests that are currently available for cytologically indeterminate FNAs. As Table 6 shows, the sensitivity of IRC is higher than that of all the five commercial molecular tests. In terms of specificity, the IRC performs superior to Afirma but inferior to ThyroSeq v2, ThyroSeq v3, ThyGenX/ThyraMIR, and RosettaGX Reveal. In the cancer prevalence range of 20–40% for ITNs, which is reported in most studies [6], the range of NPV of the IRC is 37–61% and the range of PPV is 98–99%.

## 4. Discussion

Thyroid FNAs that are classified into Bethesda III and IV are recommended for molecular tests, according to the 2015 American thyroid association management guidelines for adult patients with thyroid nodules and differentiated thyroid cancer [6]. Patients with ITNs, who implement the molecular tests, have gained benefit from avoiding the unnecessary surgery [18]. Alone with the increasing demands of the utility of molecular tests, more effective biomarkers will be detected, and more precise classifiers will be developed. Nevertheless, the limitations of the currently available commercial molecular tests confine their extensive use. Surgical intervention is still the prior option for patients with ITNs in most regions all over the world, especially for those without coverage of medicare. Some practical and cost-effective classifiers have been developed to solve this dilemma in recent years. An in vitro diagnostic gene classifier based on quantitative polymerase chain reaction (qPCR IVD), which can be performed in the hospitals equipped with qPCR, has been created and shows as a high classification capacity as the commercial molecular tests [19]. BRAF V600E mutation is also used as a predictor of thyroid malignancy in the ITN. A systematic review and meta-analysis conclude that the specificity of this marker is 100%, but the sensitivity is only 40% [20]. In addition, some biochemical parameters, such as red cell distribution width, mean platelet volume, and thyrothropin to thyroglobulin ratio, are found to help differentiating malignant and benign TNs [21–23]. In this study, we developed a classifier based on an RNA-sequencing system and seven RNA expression microarrays. The classifier will fit in different platforms, at least the platforms we used in this study, and the testing can be finished by the physicians in laboratories or by the technicians in the corresponding companies at a relatively low cost.

A powerful classifier tends to contain two essential components, distinguishing features and robust algorithm. Vast studies have focused on the immune response in the cancer microenvironment. As we know, the initiation and progression of cancer are closely associated with different immune responses in vivo, which can be used to distinguish malignant and benign TNs. It is reported that patients with malignant TNs have significantly higher neutrophil to lymphocyte ratio (NLR) than those with benign TNs (2.1 ± 0.9% *vs.* 1.7 ± 0.9%), and NLR may be useful in the differentiation of benign and malignant TNs [24]. The tumor-associated antigens expressing on thyroid cancer cells recruit various leukocytes and constitute the cancer microenvironment. On histological samples, it is observed that lymphocytic infiltration is more frequent and severe in papillary thyroid carcinoma than in multinodular goiter (82.5% *vs.* 45.0%, *P* < 0.001) and single/isolated thyroid nodule (85.6% *vs.* 71.0%, *P* < 0.001) [25]. The different IRG profile, mainly derived from the immune cells infiltrating in the TNs, can represent the different immune responses against the abnormal thyroid cells. Based on this, we used IRGs to generate a classifier for TN classification, and as we expected, the classifier showed superior discriminative capacities.

Since the distinguishing features were collected, a robust algorithm, which can not only filter and extract the useful features but also guarantee high discriminative accuracy and generalization ability, was needed. A variety of autoencoders have been used in many fields, such as feature extraction and classification. The denoising autoencoder has been employed in deep genomic feature extraction in breast cancer, and the extracted features are significantly associated with patients' clinical characteristics and outcomes [26]. Stacked sparse autoencoder has been used for cancer prediction, and the predictive performance is better than other three common algorithms, support vector machine, random forest, and neural network [27]. SDSAE, an autoencoder with three structures (stack, corruption, and sparsity), performs better in image classification than other autoencoders only with one or two structures [17].

In this study, we used an SDSAE with a softmax classifier to develop the immune-related classifier (IRC). The IRC performed well in the independent testing set, with an accuracy of 92.9%, sensitivity of 98.6%, and specificity of 58.3%. SDSAE was used in image data in the previous study [17], but it is still unknown whether it is suitable for gene data. To demonstrate the robustness of SDSAE, we compared it with other four powerful algorithms, multilayer perception, logistic regression, random forest, and support vector machine. The results of the four machine learning algorithms were not as good as SDSAE. The multilayer perception and random forest classified all the samples of the testing set into malignancy. Although the logistic regression and support vector machine both had an accuracy of 86.9% and sensitivity of 98.6%, the low specificity of 16.7% had a huge impact on them. The data we used to train the classifier were imbalanced, with 85.8% of the malignant and 14.2% of the benign. This imbalance caused the classifier to learn more of the malignant rather than the benign, resulting in a higher sensitivity but lower specificity. Interestingly, SDSAE seemed less impacted by the imbalance and performed better than the logistic regression and support vector machine. As we know, autoencoder is a good tool to extract the features of the data. We can control the feature number by adjusting the neuron number of the hidden layers. In this study, we set the neuron number of the hidden layer as 20, which indicated that the original feature number of 9377 was compressed into 20. The lower feature number relative to the sample number can reduce the risk of overfitting. This theory can explain why the classifier based on SDSAE has better performance than those based on other four machine learning. In addition, the denoising and sparse operations of SDSAE can resist the overfitting and reinforce the robustness. Although the SDSAE showed better performance than other four machine learning, the identification of benign TNs was still not as good as that of malignant TNs. More benign samples should be added to improve the IRC, despite the insufficiency of benign samples on the databases.

We compared the IRC with five commercially available classifiers. The IRC had a higher sensitivity than the five commercial classifiers. However, the specificity of the IRC was only higher than the Afirma but lower than other four classifiers. High specificity and PPV allow classifier as a “rule-in” tool to predict malignant TNs. On the other hand, a classifier with high sensitivity and NPV is a good tool to identify benign TNs. An ideal molecular test would have a high PPV similar to a malignant cytological diagnosis (98.6%) and a high NPV similar to a benign cytological diagnosis (96.3%) by the Bethesda diagnostic system [7]. Currently, no molecular tests can meet this requirement. However, in a cancer prevalence range of 20–40% for ITNs, which is reported in most studies, the IRC is qualified as “rule-out” tools, with an NPV of 98–99%, which is the same as ThyroSeq v3 and Rosseta GX Reveal.

The strength of the IRC is that they are developed based on a large amount of TN samples, which will enhance the robustness of the classifiers. Besides, gene data were tested on different platforms, indicating that physicians can test samples with diverse testing approaches from different companies. However, the TN samples are from postoperative tissue rather than FNA samples. The effectiveness of the classifiers needs validation in the cytological ITNs. A previous commercial classifier has supported the availability of the IRC classifier in cytological ITNs. ThyroSeq v3 was developed based on 238 tissue samples of TNs with an accuracy of 92.1%, and it was demonstrated to have a high accuracy of 90.9% in 175 FNA samples of ITNs [11].

## 5. Conclusion

This study developed an immune-related classifier that accurately classified malignant and benign TNs. It may benefit patients with ITNs from avoiding unnecessary surgeries. However, validation of their effectiveness on cytological ITNs is needed before clinical practice.

## Figures and Tables

**Table 1:** Testing platforms of the datasets used in this study.

Accession no.	Testing platform
TCGA thyroid carcinoma	Illumina hiSeq
E-MEXP-97	[HG-U133A] affymetrix geneChip human genome HG-U133A
E-MEXP-2442	[HG-U133_Plus_2] affymetrix geneChip human genome U133 plus 2.0
GSE27155	[HG-U133A] affymetrix geneChip human genome HG-U133A
GSE54958	[HuGene-1_0-st] affymetrix human gene 1.0 ST array [transcript (gene) version]
GSE33630	[HG-U133_Plus_2] affymetrix geneChip human genome U133 plus 2.0
GSE29315	[HG_U95Av2] affymetrix human genome U95 version 2 array
GSE65074	[HG-U219] affymetrix human genome U219 array
GSE82208	Illumina hiSeq

TCGA: The *Cancer* Genome Altas.

**Table 2:** Pathology of the samples from different datasets.

Accession no.	Papillary thyroid carcinoma	Follicular thyroid carcinoma	Anaplastic thyroid carcinoma	Hurthle cell carcinoma	Medullary thyroid carcinoma	Follicular adenoma	Hurthle cell adenomas	Hashimoto throiditis	Hyperplasias
TCGA thyroid carcinoma	564	3	—	1	—	—	—	—	—
E-MEXP-97	—	12	—	—	—	12	—	—	—
E-MEXP-2442	2	18	4	—	—	34	—	—	9
GSE27155	51	13	4	8	2	10	7	—	—
GSE54958	31	—	—	—	—	7	—	—	—
GSE33630	49	—	11	—	—	—	—	—	—
GSE29315	22	9	—	—	—	17	9	6	8
GSE65074	38	—	—	—	—	—	—	—	—
GSE82208	—	27	—	—	—	25	—	—	—

**Table 3:** Performance of the IRC based on SDSAE.

Parameter	Training set (*n* = 929)		Independent testing set (*n* = 84)	
	Value	CI of 95%	Value	CI of 95%
Proportion of malignancy	85.8%	—	85.7%	—
Area under the curve	0.887	0.855–0.920	0.785	0.638–0.931
Accuracy	92.0%	92.0–92.0%	92.9%	92.7–93.0%
Sensitivity	93.4%	91.6–95.1%	98.6%	95.9–101.3%
Specificity	84.1%	77.9–90.3%	58.3%	30.4–86.2%
Positive likelihood ratio	5.868	3.962–8.689	2.367	1.211–4.625
Negative likelihood ratio	0.079	0.060–0.104	0.024	0.003–0.177
Positive predictive value	97.3%	96.1–98.4%	93.4%	87.8–99%
Negative predictive value	67.7%	60.5–74.8%	87.5%	64.6–110.4%

IRC: immune-related classifier; SDSAE: stacked denoising sparse autoencoder; CI: confidence interval.

**Table 4:** The accuracy of the IRC in various pathological classification.

Pathology	Accuracy in training set	Accuracy in testing set
Papillary thyroid carcinoma	694/708 (98.0%)	49/49 (100%)
Follicular thyroid carcinoma	37/70 (52.9%)	11/12 (91.7%)
Anaplastic thyroid carcinoma	8/8 (100%)	11/11 (100%)
Hurthle cell carcinoma	3/9 (33.3%)	—
Medullary thyroid carcinoma	2/2 (100%)	—
Follicular adenoma	79/93 (84.9%)	7/12 (58.3%)
Hurthle cell adenomas	10/16 (62.5%)	—
Hashimoto throiditis	5/6 (83.3%)	—
Hyperplasias	17/17 (100%)	—

Data were presented as number (%). IRC: immune-related classifier.

**Table 5:** Performance of the classifiers based on the two common algorithms of machine learning.

Parameter	LR		SVM	
	Value	CI of 95%	Value	CI of 95%
Proportion of malignancy	85.7%	—	85.7%	—
Area under the curve	0.576	0.465–0.687	0.576	0.465–0.687
Accuracy	86.9%	86.6–87.2%	86.9%	86.6–87.2%
Sensitivity	98.6%	95.9–101.3%	98.6%	95.9–101.3%
Specificity	16.7%	−4.4–37.8%	16.7%	−4.4–37.8%
Positive likelihood ratio	1.183	0.917–1.526	1.183	0.917–1.526
Negative likelihood ratio	0.083	0.008–0.849	0.083	0.008–0.849
Positive predictive value	87.7%	80.5–94.8%	87.7%	80.5–94.8%
Negative predictive value	66.7%	13.3–120.0%	66.7%	13.3–120%

LR: logistic regression; SVM: support vector machine; CI: confidence interval.

**Table 6:** Comparisons of IRC with five commercial classifiers in the cancer prevalence range of 20–40% for indeterminate thyroid nodules.

Parameter	IRC (%)	Afirma (%)	ThyroSeq v2 (%)	ThyroSeq v3 (%)	ThyGenX/thyraMIR (%)	Rosseta GX reveal (%)
Sensitivity	99	92	90	98	89	98
Specificity	58	52	93	82	85	78
Positive predictive value	37–61	32–56	76–90	57–78	60–80	53–75
Negative predictive value	98–99	91–96	93–97	98–99	92–97	98–99

IRC: immune-related classifier.

## Data Availability

The datasets generated during and/or analysed during the current study are available from the corresponding author on reasonable request.

## Supplementary Materials

Supplementary Materials Supplementary material is an excel file. It includes the details of the immune-related genes we used to construct the classifier.
